# Laryngeal Eggshell Foreign Body Mimicking Persistent Laryngitis: A Case Report

**DOI:** 10.3390/reports9030239

**Published:** 2026-07-23

**Authors:** Konstantina Dinaki, Constantinos Papadopoulos, Rafail Ioannidis, Konstantinos Valsamidis, Athanasia Printza

**Affiliations:** School of Medicine, Aristotle University of Thessaloniki, 541 24 Thessaloniki, Greece; konnidin@windowslive.com (K.D.); constantine.mbg@gmail.com (C.P.); raphaioan@gmail.com (R.I.); kosvals@hotmail.com (K.V.)

**Keywords:** pediatric emergency, aspiration, laryngeal foreign body, airway, food choking, prevention, children, life- threatening, morbidity

## Abstract

**Background and Clinical Significance:** Foreign body aspiration is an important cause of morbidity and mortality in children younger than three years of age. Although laryngeal foreign bodies are uncommon, they may be life-threatening and are frequently misdiagnosed because of their variable clinical presentation. Eggshell aspiration is exceptionally rare, with only a few cases reported in the literature. We report a case of delayed diagnosis of a glottic eggshell foreign body in a toddler presenting with persistent upper airway symptoms. **Case Presentation:** A 16-month-old previously healthy girl was referred to our hospital for evaluation of persistent hoarseness and barking cough following a witnessed choking episode while eating boiled egg. The choking episode had occurred 15 days before presentation during an episode of viral upper respiratory tract infection. Initial symptoms were attributed to laryngitis and persisted despite medical treatment. Flexible laryngoscopy revealed a foreign body impacted at the glottic level, while neck radiography demonstrated a radiopaque calcified lesion corresponding to the foreign body. Microlaryngoscopy under deep sedation was performed, and an approximately 10-mm eggshell fragment was successfully removed. A small amount of granulation tissue was observed at the posterior commissure, corresponding to the site of foreign body impaction. The postoperative course was uneventful, and repeat endoscopic examination on postoperative day three demonstrated satisfactory laryngeal healing with regression of the granulation tissue. **Conclusions:** This case highlights the importance of considering a retained laryngeal foreign body in young children with persistent hoarseness or barking cough following a choking episode, even in the presence of concomitant respiratory infection. Early endoscopic evaluation is essential to avoid diagnostic delay and facilitate prompt treatment. In addition, this report emphasizes the importance of food choking prevention and caregiver education in children younger than three years of age.

## 1. Introduction and Clinical Significance

Foreign body aspiration is a common pediatric emergency and an important cause of morbidity and mortality in children younger than three years of age. In this age group, choking injuries rank among the leading causes of accidental death, and most choking events are food-related [1].

Most aspirated foreign bodies are located within the tracheobronchial tree, whereas laryngeal foreign bodies are uncommon and account for only a small proportion of airway foreign body aspirations [2,3,4]. Nevertheless, they represent a potentially life-threatening condition because even partial airway obstruction at the level of the glottis may lead to significant respiratory compromise [2,3].

The diagnosis of laryngeal foreign bodies may be challenging. Clinical manifestations are variable and depend on the size, shape, and location of the foreign body. Patients may present with stridor, dysphonia, hoarseness, barking cough, or symptoms resembling common pediatric respiratory conditions, including laryngitis, croup, or asthma. Consequently, diagnostic delay and misdiagnosis are common [5,6,7].

Several studies have emphasized that a witnessed choking episode combined with persistent upper airway symptoms should prompt suspicion of a retained laryngeal foreign body and early endoscopic evaluation [5,8]. Eggshell aspiration is exceptionally rare, with only isolated cases described in the literature [9,10,11]. We present the case of a 16-month-old girl with delayed diagnosis of a retained glottic eggshell fragment following a witnessed choking episode. This case highlights the diagnostic challenges posed by laryngeal foreign bodies that mimic common pediatric respiratory disorders and emphasizes the importance of prompt endoscopic evaluation and food choking prevention strategies.

## 2. Case Presentation

A 16-month-old previously healthy girl was referred from a secondary care regional hospital to the Pediatric Emergency Department of AHEPA University Hospital for further evaluation of a suspected airway foreign body. Following initial pediatric assessment, she was referred to the Otorhinolaryngology Department because of persistent hoarseness, barking cough, and a history suggestive of foreign body aspiration.

Approximately 15 days before presentation, the child had symptoms of an upper respiratory tract infection, similar to those experienced by her older sibling. During this period, she was fed mashed boiled egg by her grandmother and experienced a witnessed choking episode, characterized by sudden coughing followed by vomiting. The event was reported to the child’s mother, who sought medical evaluation the following day because the patient had developed persistent hoarseness and a barking cough resembling previous episodes of laryngitis.

The child was initially assessed by a private pediatrician and diagnosed with laryngitis. Oral corticosteroid therapy was prescribed for two days. As symptoms persisted, antibiotic treatment was subsequently added. Despite treatment, the patient continued to exhibit hoarseness and barking cough without significant clinical improvement.

Five days after the choking episode, the patient was evaluated at the secondary care regional hospital. Chest radiography, pulse oximetry assessment, and nebulized therapy were performed. During the following ten days, she presented twice more to the same hospital because of persistent symptoms. A second chest radiograph was obtained, oxygen saturation remained within normal limits, and additional nebulized therapy and antibiotic treatment were administered. Neither radiographic examination included the laryngeal region.

At her third presentation, persistent hoarseness and barking cough were accompanied by fever and a small amount of blood-stained saliva. Given the history of a witnessed choking episode and the lack of response to medical treatment, a retained airway foreign body was suspected. As pediatric laryngoscopic evaluation was not available locally, the patient was referred to our hospital.

**Figure 1:**
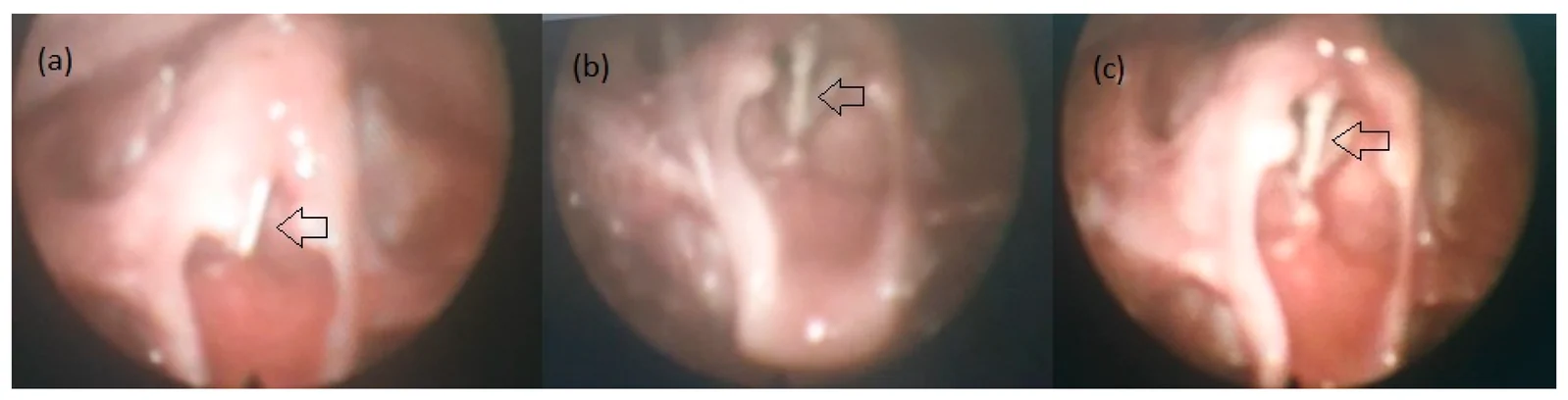
Representative flexible videolaryngoscopic views demonstrating a whitish eggshell fragment (arrow) impacted between the vocal folds at the glottic level. (**a**) Initial view of the impacted foreign body. (**b**,**c**) Additional representative views obtained from different angles during the examination.

**Figure 2:**
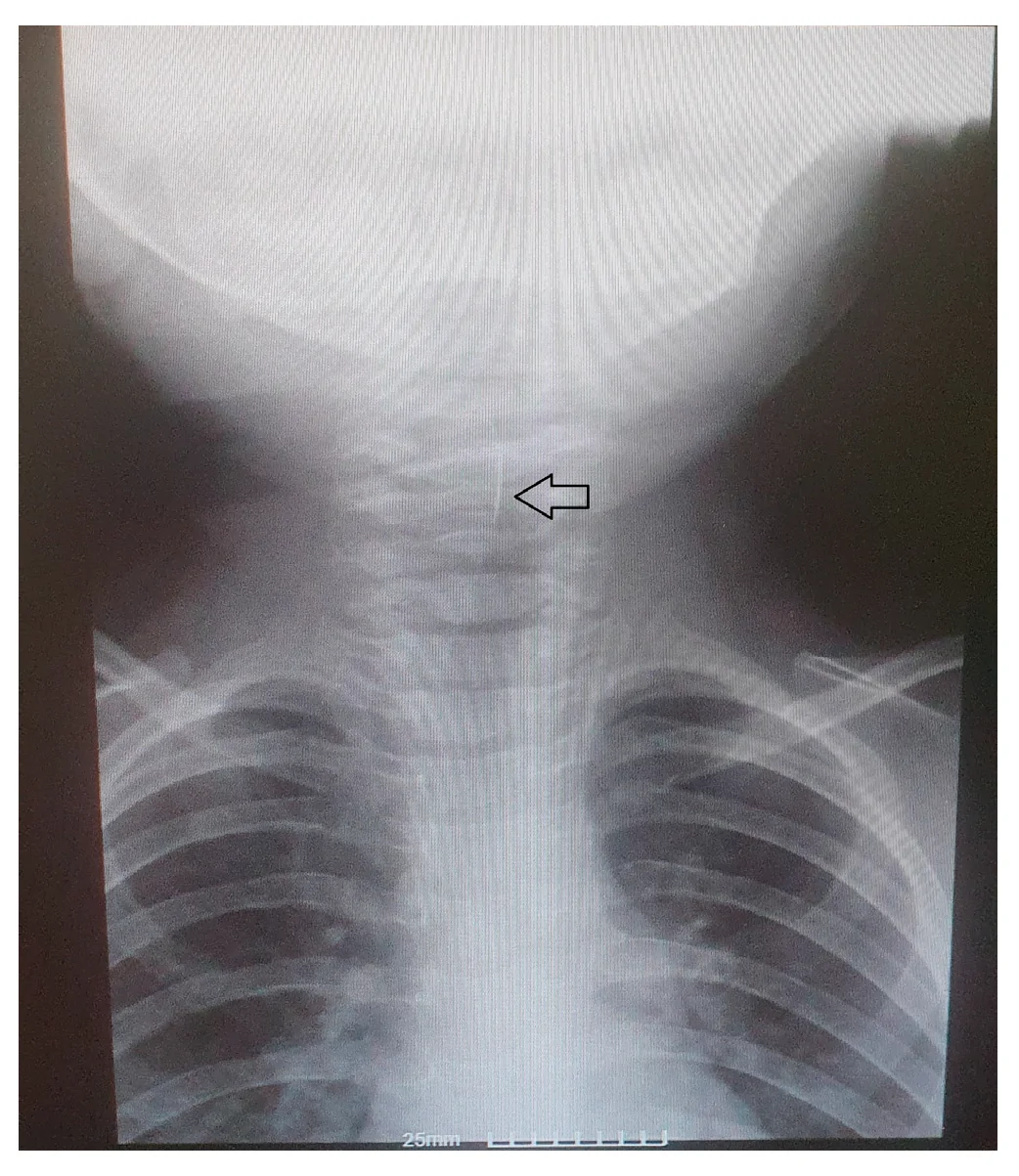
Anteroposterior neck radiograph showing a radiopaque foreign body (arrow) at the level of the larynx, later confirmed endoscopically to be an eggshell fragment.

Upon arrival, the child was hemodynamically stable and in no respiratory distress. Oxygen saturation was normal. Physical examination revealed persistent hoarseness and barking cough. Flexible videolaryngoscopy demonstrated a foreign body impacted at the glottic level (Figure 1). The preoperative endoscopic appearance of the glottic foreign body is demonstrated in Supplementary Video S1. Subsequently, anteroposterior neck radiography was performed and revealed a radiopaque foreign body at the level of the larynx (Figure 2).

**Figure 3:**
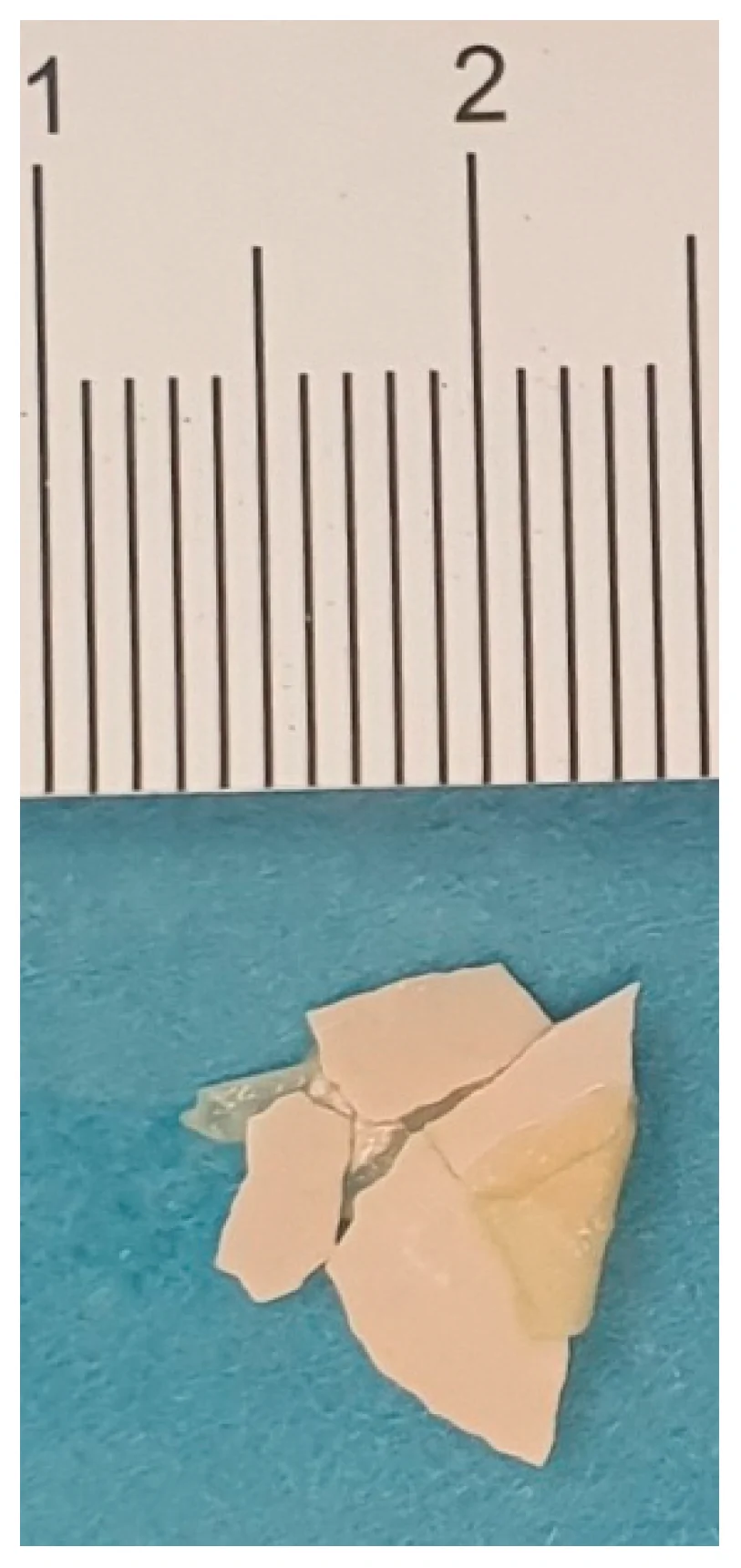
Retrieved eggshell fragment following endoscopic removal. The specimen measured approximately 10 × 12 mm and corresponded to the radiopaque lesion identified on preoperative imaging. The fragment was broken during retrieval.

The patient underwent urgent microlaryngoscopy under deep sedation using a Macintosh blade (size 1). A rigid eggshell fragment measuring approximately 10 × 12 mm was identified firmly impacted within the glottis and was successfully removed under direct visualization using grasping laryngeal forceps (Figure 3). Following removal, a small amount of granulation tissue was observed at the posterior commissure, corresponding to the site of foreign body impaction. No bleeding occurred during the procedure, and no additional pathology was identified. Recovery was uneventful.

**Figure 4:**
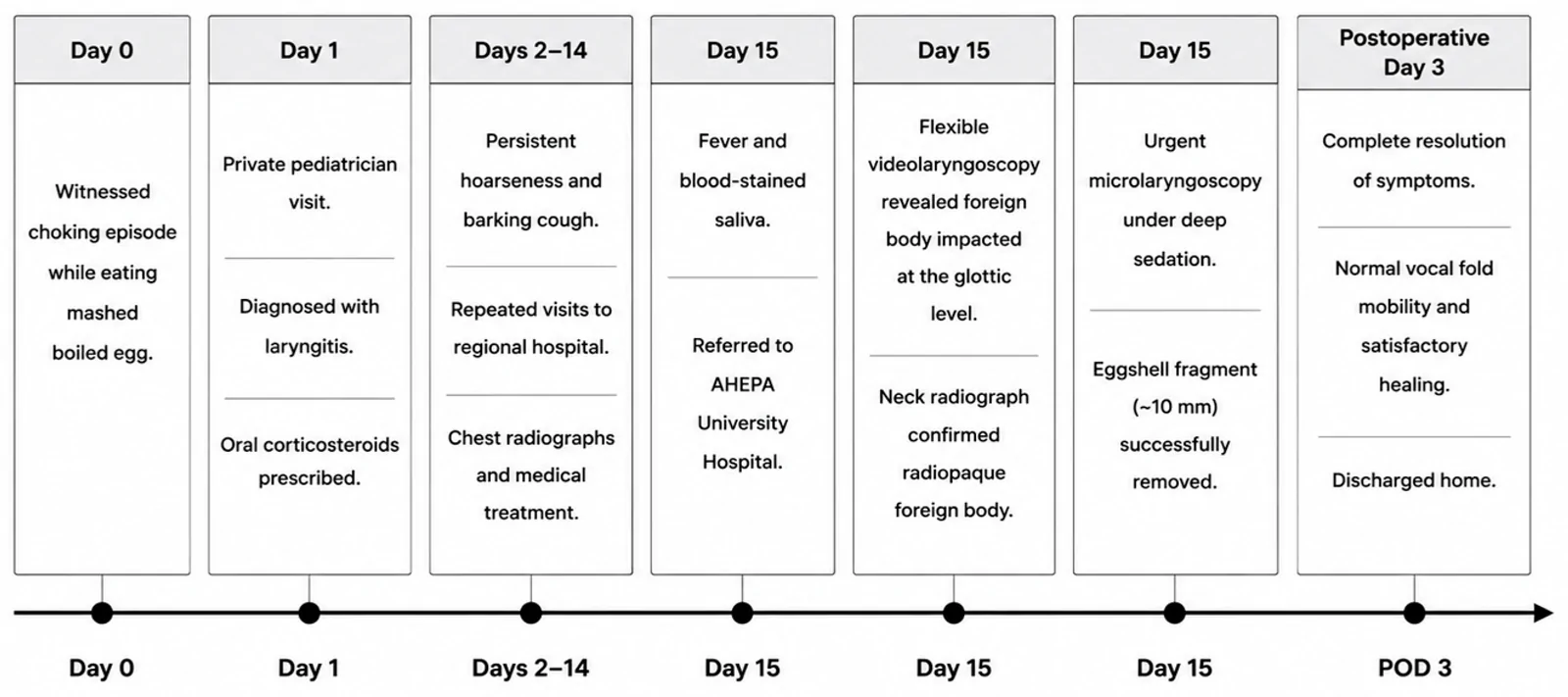
Timeline of the patient’s clinical course. Schematic overview of the patient’s clinical course, illustrating the sequence of clinical events from the witnessed choking episode through repeated medical evaluations, referral to our hospital, diagnosis of the glottic eggshell foreign body, endoscopic removal, and postoperative recovery. POD: postoperative day.

Postoperatively, the patient remained afebrile, hemodynamically stable and in no respiratory distress. She tolerated oral feeding without difficulty. Repeat flexible endoscopic examination on the third postoperative day demonstrated normal laryngeal healing without residual foreign material or significant mucosal abnormalities. The previously observed granulation tissue showed gradual regression. The patient was discharged home on the same day in good clinical condition. The chronological sequence of the patient’s clinical course is summarized in Figure 4.

## 3. Discussion

Laryngeal foreign bodies are relatively uncommon in children, accounting for only a small proportion of airway foreign body aspirations. However, despite their rarity, they represent a potentially life-threatening condition because of the narrow caliber of the pediatric airway and the possibility of acute airway obstruction [2,3].

Brama and Fearon emphasized that laryngeal foreign bodies are particularly challenging because both the clinical presentation and radiologic findings may be misleading, leading to delays in diagnosis and treatment [2]. They further observed that thin and flat foreign bodies may lodge between the vocal cords and remain in place with relatively mild respiratory symptoms despite their potentially hazardous location.

The present case illustrates this diagnostic challenge. Our patient had a witnessed choking episode while eating boiled egg during an episode of viral upper respiratory tract infection. Subsequently, she developed persistent hoarseness and barking cough, symptoms that were initially attributed to laryngitis. The coexistence of a plausible respiratory diagnosis may have contributed to diagnostic anchoring and delayed consideration of foreign body aspiration.

Chen et al., in their analysis of 35 pediatric cases of misdiagnosed laryngeal foreign bodies, highlighted that persistent symptoms such as hoarseness, cough, and dysphonia, especially when associated with a choking history, should always raise suspicion of a retained laryngeal foreign body [5]. Similarly, Cinar et al. reported a laryngeal foreign body initially misdiagnosed as bronchial asthma [6], while Bloom et al. emphasized the heterogeneous presentation of laryngeal foreign bodies and the importance of maintaining a high index of suspicion [7]. Swibel Rosenthal et al. described a chronic glottic foreign body that remained undiagnosed for nine months before being identified radiographically, further illustrating the potential for prolonged diagnostic delay [12]. Goulioumis et al. recently described a toddler with a glottic foreign body retained for a prolonged period, underlining that the severity of symptoms does not always correlate with the duration of impaction [13].

Another important aspect of our case concerns imaging and diagnostic evaluation. The child underwent two chest radiographs during the diagnostic workup, both of which failed to establish the diagnosis because they did not adequately evaluate the laryngeal region. This case illustrates an important limitation of routine chest radiography in suspected airway foreign body aspiration, as the larynx may not be included within the field of view. When persistent upper airway symptoms and a history of choking are present, dedicated neck radiographs or early endoscopic evaluation should be considered. Persistent symptoms despite medical treatment, together with the history of choking, ultimately prompted referral for endoscopic evaluation. Laryngoscopy immediately identified the foreign body, while neck radiography confirmed the presence of a radiopaque calcified structure at the glottic level. The radiopacity of the eggshell is attributable to its predominantly calcified composition, consisting mainly of calcium carbonate. However, the radiographic detectability of eggshell fragments may vary depending on their size, thickness, orientation, and superimposition with adjacent anatomical structures. This finding is consistent with previous reports demonstrating that radiologic investigations may be inconclusive and that endoscopic examination remains the cornerstone of diagnosis in suspected laryngeal foreign bodies [5,8]. Recent pediatric evidence reinforces this concept, highlighting that inconclusive radiographic findings should not delay endoscopic evaluation when clinical suspicion of foreign body aspiration remains high [14]. This case underscores the importance of maintaining a high index of suspicion and proceeding promptly to laryngeal endoscopic evaluation when symptoms persist despite appropriate medical treatment, as early diagnosis and removal of the foreign body may prevent potentially life-threatening airway complications, a recommendation that is also supported by recent pediatric clinical experience [15].

Eggshell aspiration is exceptionally rare. Irani described the case of a two-year-old girl with an eggshell fragment wedged in the glottis after a choking episode during breakfast [10]. The child initially had no stridor or symptoms except for spasmodic cough, and the diagnosis became apparent only after persistent symptoms developed. Naveh et al. subsequently reported two infants with eggshell aspiration, one with a laryngeal and one with a tracheal foreign body, emphasizing both the diagnostic difficulty and the importance of careful radiographic evaluation [11]. More recently, Lapeña reported aspiration of an embryonated duck eggshell in a one-year-old boy presenting with chronic cough and dysphonia, initially treated as pneumonia before laryngoscopy established the diagnosis [9]. The present case adds to the limited literature regarding eggshell aspiration and demonstrates that even small fragments of eggshell may become firmly impacted at the glottic level and cause persistent symptoms despite relatively preserved respiratory status.

Beyond the diagnostic aspects, this case highlights the importance of food choking prevention in infants and toddlers. Choking injuries are among the leading causes of accidental death in children younger than three years of age, and most of them are food-related [1]. Recent public health recommendations emphasize that many choking episodes are preventable through age-appropriate feeding practices, careful food preparation, and close adult supervision during meals [1,16,17]. Although cooked egg is considered an appropriate complementary food and is commonly introduced during early infancy, complementary foods should be introduced according to the child’s developmental readiness to safely manage different food textures and consistencies [18]. Foods should be appropriately prepared to minimize choking risk, while caregivers should receive education regarding safe feeding practices and potentially hazardous foods [1,16,17]. Parental education regarding age-appropriate feeding practices and feeding safety is essential during early childhood, as caregiver behaviors and feeding practices substantially influence children’s eating habits and feeding safety [18]. Increased awareness among both parents and healthcare professionals may contribute not only to preventing aspiration events but also to earlier recognition and treatment when aspiration occurs. Recent studies have highlighted persistent gaps in knowledge regarding pediatric choking recognition and management among healthcare trainees, while also suggesting that visual educational tools may improve the acquisition of first-aid skills, further emphasizing the need for continuous education of both caregivers and healthcare professionals [19,20].

## 4. Conclusions

Laryngeal foreign bodies are rare but potentially serious causes of persistent upper airway symptoms in young children. A witnessed choking episode followed by persistent hoarseness, barking cough, or failure to respond to conventional treatment should prompt consideration of a retained laryngeal foreign body, even in the presence of a concomitant respiratory infection. Early endoscopic evaluation remains essential for timely diagnosis and management. Finally, this case underscores the importance of caregiver education and preventive strategies aimed at reducing food-related choking injuries during early childhood.

## Data Availability

The original contributions presented in this study are included in the article. Further inquiries can be directed to the corresponding author.

## Supplementary Materials

The following supporting information can be downloaded at https://www.mdpi.com/article/10.3390/reports9030239/s1, Supplementary Video S1. Preoperative endoscopic visualization of the glottic eggshell foreign body in a 16-month-old girl presenting with persistent hoarseness and barking cough following a choking episode.
